# Effects of home-based resistance training and neuromuscular electrical stimulation in knee osteoarthritis: a randomized controlled trial

**DOI:** 10.1186/1471-2474-13-118

**Published:** 2012-07-03

**Authors:** Robert A Bruce-Brand, Raymond J Walls, Joshua C Ong, Barry S Emerson, John M O’Byrne, Niall M Moyna

**Affiliations:** 1 Cappagh National Orthopaedic Hospital, Finglas, Dublin 11, Ireland; 2 Centre for Preventive Medicine, Dublin City University, Dublin, Ireland

## Abstract

**Background:**

Quadriceps femoris muscle (QFM) weakness is a feature of knee osteoarthritis (OA) and exercise programs that strengthen this muscle group can improve function, disability and pain. Traditional supervised resistance exercise is however resource intensive and dependent on good adherence which can be challenging to achieve in patients with significant knee OA. Because of the limitations of traditional exercise programs, interest has been shown in the use of neuromuscular electrical stimulation (NMES) to strengthen the QFM. We conducted a single-blind, prospective randomized controlled study to compare the effects of home-based resistance training (RT) and NMES on patients with moderate to severe knee OA.

**Methods:**

41 patients aged 55 to 75 years were randomised to 6 week programs of RT, NMES or a control group receiving standard care. The primary outcome was functional capacity measured using a walk test, stair climb test and chair rise test. Additional outcomes were self-reported disability, quadriceps strength and cross-sectional area. Outcomes were assessed pre- and post-intervention and at 6 weeks post-intervention (weeks 1, 8 and 14 respectively).

**Results:**

There were similar, significant improvements in functional capacity for the RT and NMES groups at week 8 compared to week 1 (p≤0.001) and compared to the control group (p < 0.005), and the improvements were maintained at week 14 (p≤0.001). Cross sectional area of the QFM increased in both training groups (NMES: +5.4%; RT: +4.3%; p = 0.404). Adherence was 91% and 83% in the NMES and RT groups respectively (p = 0.324).

**Conclusions:**

Home-based NMES is an acceptable alternative to exercise therapy in the management of knee OA, producing similar improvements in functional capacity. Trial registration: Current Controlled Trials ISRCTN85231954

## Background

Knee osteoarthritis (OA) is a leading cause of chronic disability in people over the age of 50 [1]. Regular exercise is associated with significant improvements in pain and disability in patients with symptomatic knee OA, and international guidelines recommend exercise in the first-line management of this condition [2-5].

Quadriceps femoris muscle (QFM) weakness is a feature of knee OA and many exercise programs place particular emphasis on strengthening this muscle group [6]. Traditional supervised resistance exercise is, however, labour-intensive, time-consuming and often logistically difficult for patients. The effectiveness of such programs is dependent on good adherence and this can be challenging to achieve and maintain for patients with significant knee OA. Furthermore, in severe knee OA, deficits in volitional muscle activation contribute more to quadriceps weakness than muscle atrophy, and this may limit the effectiveness of volitional training programs [7].

Because of the limitations of traditional exercise programs, interest has been shown in the use of neuromuscular electrical stimulation (NMES) to strengthen the QFM. NMES is the application of transcutaneous electrical current to elicit involuntary muscle contractions. A few small studies have demonstrated benefits of NMES in the conservative management of knee OA, and in the prehabilitation and rehabilitation of knee arthroplasty patients [8-11].

This pilot study attempts to address some of the gaps in our understanding of the role of exercise in the management of knee OA by comparing NMES, resistance training (RT) and standard care in subjects with moderate to severe knee OA. To our knowledge no prior studies have compared a home-based NMES program to a home-based exercise program for subjects with knee OA. We examined the outcomes of function, self-reported disability, compliance, QFM strength and cross-sectional area (CSA). Our hypothesis was that a home-based NMES program would provide similar benefits to a home-based exercise program in these outcome measures.

## Methods

### Trial design

This was a single-centre, single-blind, prospective randomised control trial.

### Participants

Eligible participants were patients of Cappagh National Orthopaedic Hospital, resident in the Greater Dublin area, aged 55–75 years, with symptomatic, moderate to severe knee OA. Participants were recruited from the arthroscopy database and knee arthroplasty waiting list. Patients were eligible to participate if they had been diagnosed arthroscopically with grade 3 or 4 OA on the Outerbridge scale within the last 2 years, or were placed within the last 6 months on the waiting list for knee replacement surgery with the indication of OA, confirmed radiographically with Kellgren-Lawrence severity grades of 3 or 4.

Exclusion criteria were medical co-morbidities precluding participation in an exercise program, implanted electrical devices, neurological disorders, inflammatory arthritis, non-ambulatory status, significant cognitive impairment, participation in an exercise program within the last 6 months, involvement in a previous similar study, anticoagulant therapy, and recent or imminent surgery (within 3 months).

Ethical approval was obtained from the Cappagh National Orthopaedic Hospital Research Ethics Committee, and the procedures followed were in accordance with the Helsinki Declaration of 1975, as revised in 2000. The trial was registered with Current Controlled Trials (ISRCTN85231954).

All patients meeting the eligibility criteria were contacted, given verbal and written information on the trial, and invited to participate. Consenting patients underwent a clinical examination to confirm eligibility, and provided written informed consent.

### Interventions

Subjects were randomised to a 6-week home-based resistance-training (RT) exercise program, a 6-week home NMES program (NMES) or a control group (C) receiving standard care.

### Resistance training group

The RT group undertook 3 home-based training sessions per week for 6 weeks. Each session was approximately 30 min in duration and was separated by a minimum of 36 h. Two of the three weekly sessions were supervised by an exercise specialist to ensure that each exercise was performed using the correct technique. Both lower limbs were trained using the following exercises in this order: knee presses, bottle knee presses, extended leg raises, leg extensions, wall squats and hamstring curls. The exercise regimen comprised 3 sets of 10 repetitions for each of the 6 exercises. Each set was performed bilaterally, starting with the less affected limb (except for the wall squats which exercised both limbs simultaneously). The logbook supplied to RT participants contained detailed instructions on the prescribed exercises.

The knee press exercise required the subject to lie supine on a bed or floor, actively dorsiflex the ankle and toes and contract the QFM of the exercising limb while pushing the knee posteriorly into the bed or floor for at least 5 s. The bottle knee press was performed in an identical manner with the addition of a water-filled 2 L plastic bottle placed under the exercising knee, while the contralateral knee was kept extended. The bottle provided resistance to knee extension. For the extended leg raise, the subject lay supine with the resting limb flexed at the knee and the resting foot flat on the floor or bed. Keeping the knee of the exercising limb extended and the ankle and toes dorsiflexed, this limb was lifted 20 cm off the bed or floor and held for at least 5 s before slowly lowering it to the surface. The leg extension exercise required the subject to sit back in a chair holding onto the sides of the seat for support, fully extend the knee while keeping the ankle dorsiflexed, and hold this position for at least 5 s. For the wall squat exercise the subject stood about 60 cm in front of a wall and leaned against it, with feet pelvis-width apart and slightly externally rotated, then flexed the knees and slowly slid down the wall until significant contraction was felt in the QFM, before sliding back up the wall to the starting position. The hamstring curl required the subject to stand with the hands on a table or back of chair for support, and flex one knee so that the heel approximated the buttock as far as possible.

Resistive bands (Thera-Bands^®^) were used to provide resistance for the extended leg raises, leg extensions and hamstring curls by wrapping the band once around each ankle, leaving about 20 cm of band between the ankles.

Subjects kept a logbook and recorded their rating of perceived exertion (RPE) using the 15 point Borg scale, and pain scores using a 0–10 analogue scale for each session. Subjects were encouraged to increase the intensity of the exercise if they consistently obtained RPE scores below 14 with pain scores below 3. This was achievable by holding the muscle contraction for a longer period of time, or by using higher resistance bands.

### NMES group

The NMES group undertook a single 20 min unsupervised NMES session of the affected QFM, 5 days per week for 6 weeks. Bilateral NMES training was undertaken if the subject suffered from significant bilateral knee OA (such that the less affected knee contributed significantly to functional disability). Subjects were instructed to train at the same time of the day to ensure adequate muscle recovery. Each stimulation cycle comprised a 10 s contraction period and a 50 s relaxation period, excluding the 1 s ramp-up and 0.5 s ramp-down. This provided a total contraction time of 3 min 20 s in each 20 min session.

A portable, battery-powered garment-based NMES stimulator was used (Kneehab^®^, Bio-Medical Research Ltd, Galway, Ireland). The device utilises two channels and a “multipath” system of stimulation to coordinate muscle contraction. The stimulator produces a symmetrical bi-phasic square waveform, with a maximum root mean square output current of 18 mA and an output frequency of 50 Hz. Pulse width changes dynamically during the stimulation cycle between 100–400 μs. Four reusable adhesive hydrogel electrodes (Axelgaard, Fallbrook, CA), having surface areas of 194 cm^2^, 83 cm^2^, 74 cm^2^ and 66 cm^2^ respectively, are attached to the deep surface of the garment and conduct impulses to the vasti and rectus femoris muscles.

Subjects were instructed to perform the NMES training in the seated position with the knee flexed to 60 degrees, the foot flat on the floor, and the toes pressed against a wall to achieve isometric muscle contraction. They were encouraged to always increase the stimulation intensity to the maximally tolerated level. Comprehensive training on device usage was provided prior to the NMES program. It was explained to them that they would become more tolerant of the discomfort associated with the stimulation with repeated usage, and were expected to progressively increase their maximally tolerated intensity level during the course of the 6 week program. Subjects kept a logbook and recorded the date, duration, RPE and pain score for each session.

### Control group

Subjects in the control group received standard care. This included OA education, weight loss, pharmacologic therapy, and physical therapy. They were not discouraged from maintaining their existing level of activity.

Subjects in all 3 groups were advised to maintain any pre-existing treatment of their OA such as pharmacologic therapy.

### Outcomes

The primary outcome measure was objective functional capacity, assessed using a 25 m walk test, a repeated chair rise test, and a stair climb test. Secondary outcome measures were Western Ontario McMaster Universities osteoarthritis index (WOMAC) physical function, pain and stiffness scores, Short Form Health Survey (SF-36) physical health and mental health scores, peak isometric and isokinetic quadriceps torque and quadriceps CSA.

Functional capacity, self-reported disability, peak isometric and isokinetic quadriceps torque were assessed at baseline/week 0 (familiarisation), week 1 (pre-intervention), week 8 (post-intervention) and week 14 (6 weeks post-intervention). Quadriceps CSA was assessed in the intervention groups at week 1 and week 8. Familiarisation testing, conducted in an identical manner to subsequent dynamometry and functional testing, was used to reduce the effect of improved technique on the results and minimise bias when comparing pre- and post-intervention results.

The 25 m walk test, repeated chair stand test, and stair climb test were performed in this order. Each test was performed 3 times with a 30 s rest period between each trial. A 5 min rest period was provided between the different functional tests. Subjects were instructed to perform each test as quickly as possible, but in a controlled and safe manner. The fastest time achieved for each test was recorded. Standardised verbal instructions and encouragement were given.

The 25 m walk test was undertaken on a pre-marked 35 m indoor corridor. A 5 m lead-in and 5 m end zone were included to minimise the effect of variable acceleration and deceleration. Timing began when the subject’s leading foot crossed the 5 m mark, and stopped when the trailing heel crossed the 30 m mark. If the subject used a mobility aid for normal ambulation, he/she was required to perform all the walk tests with the aid.

For the repeated chair stand test, the subject was required to perform 3 consecutive cycles of standing with fully extended knees immediately followed by sitting with shoulders touching the backrest. A straight-back padded chair with armrests and adjustable leg height was used. The leg height was adjusted and recorded so that the subject’s knee was flexed at 90° in the seated position. Some subjects performed each chair stand test with arms folded across their chest while others used the armrests for all assessments.

The timed stair climb test was performed on an indoor stairwell with 11 steps. Subjects were required to ascend the stairs, turn on the landing, and descend as quickly as possible, without skipping any steps. The handrail could be used if required.

Isometric and isokinetic quadriceps peak torque were measured using a Biodex dynamometer (Biodex Multi-Joint System 3, Biodex Medical Systems, Inc. Shirley, New York, USA). Subjects were seated in the dynamometer in an upright position with 90° of hip flexion. Both limbs were tested, beginning with the unaffected or least affected limb. For familiarisation and warm-up purposes, subjects were asked to perform a set of 3 progressive sub-maximal isometric contractions and a transient maximal contraction, attempting to extend the knee against the immobile force pad. The subsequent isometric test comprised 3 consecutive maximum volitional isometric contractions (MVIC) of the quadriceps muscles at 60° of knee flexion. Each contraction was of 5 s duration, with a rest period of 50 s between each. The Biodex software (Revision 3.30) allowed automation of the timing of this procedure. Standardised verbal instructions and encouragement were given. The maximum force generated over the 3 trials was recorded as the peak torque. The process was repeated on the (more) involved limb after a 3 min rest period.

Isokinetic peak torque was then assessed for each limb. The dynamometer was first calibrated with the range of motion of the knee under test. Subjects were then required to perform 5 contiguous maximal concentric isokinetic repetitions (extension and flexion) at 60°/s. The peak torque value for extension was recorded. The process was repeated on the (more) involved limb after a 3 min rest period.

The WOMAC index is an OA-specific health survey, comprising 24 questions scored from 0 to 4 (best to worst respectively). The index is subcategorised into pain (scored 0–20), stiffness (0–8), and physical function (0–68). The SF-36 is a 36 item, generic, non-disease-specific health survey. Aggregate scores are given for physical and mental health, both scored from 0 to 100 with a higher score indicating better health. The reliability and validity of the WOMAC and SF-36 are well established [12,13].

Adherence was defined as the percentage of prescribed sessions completed, and was calculated for all subjects that commenced RT or NMES training. Calculations were based on the data recorded in the logbooks. The reliability of logbook recording was examined for the NMES group by comparing the device’s internal log to the patient logbook at the end of the 6-week intervention.

Magnetic resonance imaging (MRI) was used to measure quadriceps CSA. A Philips Gyroscan Intera 1.5 T scanner (Philips Medical Systems, The Netherlands) was used to scan both thighs. A slice thickness of 4 mm and slice gap of 0.4 mm was used with an echo time of 0.1 s and relaxation time of 3.0 s. Subjects were scanned in the supine position. A coronal scouting scan was used to establish the level of the mid-thigh using the greater trochanter and knee joint line as anatomical marks. Twelve T2-weighted axial images were produced, centred on the mid-thigh level. Each image had a field of view of 30 cm, and comprised a 256 x 256 pixel matrix. A single clinician, blinded to group assignment, analysed the images and determined the CSA using EasyVision software (Philips Medical Systems). Mid-sequence images from each subject’s baseline and post-intervention scans were compared, and anatomical markers used to ensure comparisons were made at identical anatomical levels. The software was used to manually delineate the QFM on these matched central axial images and to compute the enclosed CSA (in mm^2^).

### Randomisation

Patients were recruited by the first investigator (RBB). Group assignment took place when all eligible, consenting subjects had passed a clinical examination. This was undertaken by an investigator with no clinical involvement in the trial.

A stratified randomisation technique was employed to ensure that gender and age distribution was similar in each group. Each subject was matched with 2 participants of the same gender and similar age. Each group of three was then randomly assigned to one of the experimental conditions using computer-generated random numbers.

### Blinding

Whereas patients and exercise specialists were aware of the allocated intervention, outcome assessors and data analysts were kept blinded to the allocation. Patients were repeatedly reminded not to disclose their intervention to outcome assessors.

### Statistical methods

Statistical analyses were performed using SPSS Version 17. Data was checked for normality using the Shapiro-Wilks test and by observation of Q-Q plots. Data from the familiarisation phase was excluded from analysis.

A one-way analysis of variance (ANOVA) was used to evaluate potential group differences in baseline characteristics (age, gender, height, body mass index and functional capacity). A mixed-design repeated measures ANOVA was used to test for main effects of group assignment over time (weeks 1, 8 and 14). Where data violated the assumption of sphericity, the Greenhouse-Geisser correction was used. The level of significance was set at 5%, with Bonferonni adjustment made for multiple comparisons. Results are expressed as mean value ± standard deviation (SD).

## Results

**Figure 1:**
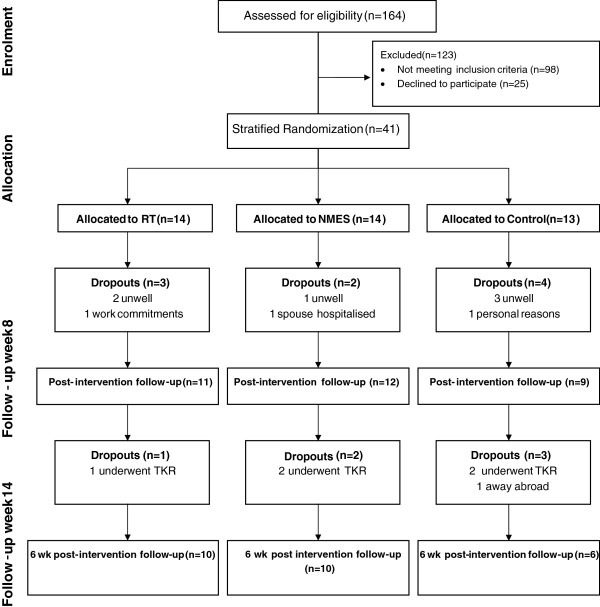
Participant flow.

Figure 1 illustrates the participant flow. A total of 164 participants were assessed for eligibility. 98 did not meet the inclusion criteria (38 were medically unfit, 22 were due for surgery within 3 months, 16 had had surgery within the last 3 months, 5 were on oral anticoagulants, 4 had inflammatory arthritis, 4 were uncontactable, 3 were away for a substantial part of the study, 2 had neurological disease, 2 were currently involved in an exercise program and 2 were in a recent similar study). Overall, 41 of 66 eligible participants underwent stratified randomisation, 32 of 41 (78%) completed post-intervention testing and 26 (63%) were available for assessment at week 14.

Baseline characteristics are presented in Table 1. There were no significant differences between the 3 groups in any of the measured characteristics.

**Table 1:** Baseline characteristics

	**Experimental condition**		
	**NMES (n = 10)**	**RT (n = 10)**	**Control (n = 6)**
Female: Male	4:6	4:6	3:3
Age (years)	63.9 ± 5.8	63.4 ± 5.9	65.2 ± 3.1
Height (m)	1.59 ± 0.10	1.60 ± 0.07	1.62 ± 0.15
Body mass index (kg/m^2^)	33.7 ± 5.6	33.9 ± 8.3	31.7 ± 4.1
Walk test (s)	15.43 ± 3.61	16.81 ± 3.39	16.64 ± 3.64
Chair Rise Test (s)	7.37 ± 2.05	8.33 ± 1.45	8.16 ± 2.23
Stair Climb Test (s)	14.31 ± 5.11	15.12 ± 5.32	13.27 ± 5.36

Values are mean ± standard deviation.

NMES, Neuromuscular Electrical Stimulation Group; RT, Resistance Training Group.

### Functional tests

Post hoc analysis was adjusted for multiple testing using a Bonferroni correction of p = .05/9 = 0.005). Results of all outcome measures are summarised in Table 2. Performance in the 25 m walk test, stair climb test and chair rise test improved (p≤0.001) in the RT and NMES groups at week 8 compared to week 1, and the improvements were maintained in both groups at week 14. There were no significant intergroup differences in the functional tests between the RT and NMES groups at any time point, but both therapy groups showed significant improvements relative to the control group at weeks 8 and 14 (p < 0.005). Functional test scores did not change significantly in the control group at week 8 or 14 compared to week 1.

**Table 2:** Outcome measures

	**NMES (n = 10)**			**RT (n = 10)**			**Control (n = 6)**		
	**Week 1**	**Week 8**	**Week 14**	**Week 1**	**Week 8**	**Week 14**	**Week 1**	**Week 8**	**Week 14**
Walk Test (s)	15.43±3.61	13.85±3.79*	13.08±4.08*	16.81±3.39	14.07±3.40*	14.50±3.71*	16.64±3.64	16.30±3.58	11.55±4.10
Chair Rise Test (s)	7.37±2.05	6.35±1.46*	5.79±1.20*	8.33±1.45	5.97±0.58*	6.18±1.05*	8.16±2.23	8.01±2.17	5.20±1.11
Stair Climb Test (s)	14.31±5.11	11.87±4.21*	12.48±6.44*	15.12±5.32	11.78±4.54*	11.84±5.03*	13.27±5.36	13.06±5.84	9.63±2.99
WOMAC Physical Function	41.04±11.60	33.88±12.66	31.50±12.63*	31.68±12.92	33.91±12.91	31.50±14.40	31.67±17.95	26.11±15.33	21.67±18.90
WOMAC Pain	11.50±3.50	8.88±3.29*	8.50±2.72	11.05±3.02	10.78±4.31	9.60±4.14	9.00±3.65	8.33±4.36	8.33±4.08
WOMAC Stiffness	5.17±2.17	3.92±2.15	4.10±2.18	5.23±0.68	4.73±1.74	4.45±2.24	5.22±1.72	4.11±1.83	4.00±2.37
SF-36 Physical Health	39.25±16.95	50.50±16.60*	47.60±10.73	39.73±16.51	50.00±23.12	53.20±25.09	51.78±24.34	56.00±24.74	67.83±21.71
SF-36 Mental Health	60.67±26.45	70.67±21.84	65.40±12.98	56.36±21.91	66.64±20.36	65.30±24.91	62.00±25.41	65.00±27.77	70.50±22.40
Isometric Peak Torque (Nm)	88.16±31.21	92.59±36.66	86.65±31.21	82.55±22.39	87.16±17.84	86.87±21.44	92.99±47.89	100.30±32.80	97.35±44.76
Isokinetic Peak Torque (Nm)	67.33±19.42	71.37±23.89	66.36±20.18	64.52±21.74	71.79±17.27	70.91±21.83	75.10±28.45	75.91±28.83	77.80±47.45
Quadriceps CSA (mm^2^)	4061±721	4279±784*		4335±610	4521±651*				

Values are mean **±** standard deviation.

* P significant vs. week 1 (P < 0.0056).

NMES, Neuromuscular Electrical Stimulation Group; RT, Resistance Training Group; WOMAC, Western Ontario McMaster Osteoarthritis index.

SF-36, Short Form 36; CSA: Cross-sectional area.

### Self-reported disability

There were no between-group differences in self-reported disability after therapy. The only significant intra-group changes were improvements in WOMAC pain score between week 1 and week 8 for the NMES group (p = 0.004); WOMAC physical function for the NMES group at week 14 relative to week 1 (p = 0.004), and an improved SF-36 physical health for the NMES group at week 8 relative to week 1 (p = 0.005).

### Quadriceps peak torque

There were no between-group or intra-group differences in isokinetic or isometric quadriceps peak torque after therapy (weeks 8 and 14 relative to week 1). There was however a trend towards an increase in both isokinetic and isometric strength for both training groups at week 8 relative to week 1.

### Quadriceps cross-sectional area

Quadriceps CSA was greater (p < 0.005) in the NMES and RT groups at week 8 than week 1 (+5.4% for NMES, +4.3% for RT). There was no difference in quadriceps CSA between the two training groups at week 8 (p = 0.404).

### Adherence

Adherence to the prescribed 6-week intervention was not significantly different between the training groups (NMES 91.3%, RT 83.3%, P = 0.324). Comparisons of NMES logbooks to the stimulator’s internal log at the end of the intervention period showed complete concordance. The mean RPE values were similar for the NMES group (15 ± 1.9) and the RT group (15 ± 1.5).

## Discussion

To date, relatively few studies have examined the use of NMES in the management of knee OA and there is a notable dearth of studies comparing this modality to more traditional resistance training programs in subjects with knee OA.

Exercise dosage is a function of frequency, intensity and program duration and a range of these variables have been used in the reviewed literature. No optimal dosage has been established for either resistance training or NMES [4,14]. Our choice of training frequency and intensity for each modality was based on careful consideration of what would achieve significant improvements in the outcome measures while achieving high levels of adherence.

Our RT frequency of 3 sessions per week was based on the large Fitness Arthritis and Seniors Trial (FAST) study, which achieved 70% compliance over a relatively lengthy trial lasting 18 months [15]. Frequent contact was maintained between exercise leaders and participants during the home-based phase of the FAST study, and this may have played an important part in the good compliance achieved [16]. We replicated this strategy in our study. Our RT exercise program was designed in collaboration with a senior physical therapist, using exercises routinely prescribed for patients with knee OA.

For our NMES protocol, we used a 50 Hz excitatory frequency and a 10 s on / 50 s off duty cycle, as these values were most commonly employed in the studies included in the systematic review by Bax *et al.* in 2005 [14]. We used a maximally tolerated stimulus intensity because it is easily implemented, and increases NMES dosage. Our NMES training frequency of 5 times per week was based on the benefits and high adherence achieved with this frequency for subjects with knee OA in the studies by Walls *et al.* and Durmus *et al.*[9,10]. Unlike the RT group, we did not supervise or maintain frequent contact with the NMES group, as excellent compliance has been demonstrated in unsupervised, home-based NMES quadriceps training in advanced OA [10].

We found that 6 weeks of NMES or RT exercise resulted in significant improvements in functional performance in patients with moderate to severe knee OA. The improvements in functional capacity were maintained for an additional 6 weeks after the NMES or RT programs. These functional improvements were achieved despite the fact that increases in knee extensor strength and disability scores did not reach significance compared to the control group. We did find significant intra-group improvements in WOMAC pain and SF-36 physical health for the NMES group, and a trend towards significant intra-group improvements (p < 0.03) in SF-36 physical and mental health for the RT group at the end of therapy.

Similar improvements in functional tests were found in 50 women with knee OA in response to 4 weeks of NMES and biofeedback-assisted exercise [9]. In contrast to our findings, they found significant increases in QFM strength and self-reported disability scores in both training groups.

Talbot *et al*. compared the effects of a 12-week NMES intervention program in patients with symptomatic knee OA, and found faster walking pace, quicker chair rise and decreased pain despite only modest (9%) improvements in leg extensor strength [8]. Although they used a longer intervention period, training was performed 3 days per week, and at lower intensities than the present study.

The Fitness Arthritis and Seniors Trial (FAST) study of 439 subjects with knee OA found that resistance training resulted in significantly better scores on performance measures and physical disability compared to a health education group [15]. Although the training period was 18 months in duration, there was no difference in QFM extension strength between the two experimental groups. Similarly, van Baar *et al.* found a small to moderate improvement in pain but failed to find an increase in knee extension strength in patients with OA after a 12-week exercise program, and a recent study by Palmieri-Smith *et al.* found no increase in quadriceps strength following a 4 week NMES program for 30 women with radiographic evidence of mild or moderate OA [17,18].

Buchner *et al.* showed that the relationship between strength and function is nonlinear. Small changes in physiological capacity may have substantial effects on performance in frail adults, while large changes in strength can have little or no effect on daily function in healthy adults [19]. This phenomenon may partly explain the disparate or nonlinear correlation between strength and function in the present and abovementioned studies. We did find a trend towards an increase in QFM strength for both training groups at the end of the therapy period. A larger study may show such strength increases reach significance, even if they are not proportional to the functional benefits.

A notable limitation of our study was not including measures of voluntary activation such as electromyography or twitch interpolation in order to distinguish between neuromuscular recruitment factors versus intrinsic muscle tissue or cellular factors. It would also have been useful to measure the percentage of maximal voluntary contraction (MVC) obtained with NMES during the course of the study. Talbot *et al*. found that subjects with symptomatic knee OA tolerate 30-40% MVC and the average training intensity in their study was 22% of MVC [8]. Qualitatively, patients in the current study reported an ability to increase the training intensities significantly after the first few days of training, with slower gains thereafter. This is in keeping with well-reported tolerance or accommodation to NMES [8,20]. All subjects in the NMES group in the current study were able to reach motor-threshold throughout the training program.

There is evidence that self-reported measures of physical function do not always correlate closely with objective measures of the ability to perform activities [21]. Patients’ perception of their ability to move around is influenced by pain and exertion. This study showed signicant improvements in functional performance for both training groups without consistent improvements in self-reported disability. This may be explained by the subjects’ perception of functional changes being clouded by inadequate gains in pain and overall health.

The validity of various functional tests such as walk tests and stair climb tests have been established for subjects with knee OA, although there isn’t a consensus for parameters such as the distance walked or number of stairs used. A systematic review of performance-based measures for patients with hip and knee OA showed intraclass correlation coefficients (ICCs) ranging from 0.78 to 0.99 for various walking tests, and ICCs of 0.95 and 0.98 for the two up and go tests included [22]. Kennedy et al. analysed physical performance measures of 177 patients of mean age 63.7 +/- 10.7 years with hip and knee OA awaiting arthroplasty and showed an ICC for test-retest reliability of 0.91 (0.81,0.97) for a similar walk-test, and an ICC of 0.90 (0.79, 0.96) for a similar stair climb test [23]. Their timed up and go test, with an ICC of 0.75 (0.51,0.89) differed from our timed chair rise test in that they incorporated a 3 m walk and turn element.

The percentage increases in quadriceps CSA in the present study (5.4% for NMES, 4.3% for RT) is lower than the 6% increase reported by Gondin *et al.*, following 8 weeks of NMES in healthy volunteers, and the 9.3% increase in CSA found by Frontera *et al.*, after 12 weeks of RT training in healthy elderly men [24,25]. Unlike the current study, both these studies found associated gains in QFM torque.

We find the increase in CSA in the training groups in the current study surprising and difficult to explain given the absence of significant change in QFM torque. It may be that our small study concealed a true associated strength gain that a larger sample size would have revealed. Alternatively, it may reflect a complex relationship between muscle size and strength in this cohort. NMES is capable of producing a range of neural adaptations within the central nervous system [20]. Decreases in voluntary muscle activation may negate increases in muscle size. Many studies have shown increased voluntary muscle activation with NMES and RT programs, although most examined healthy subjects [24,26]. Zory *et al.* found that QFM MVC was unchanged after 4 weeks of NMES in healthy men because of the interplay of increased activation and impaired muscle contractility [27]. Deley *et al.* found decreases in voluntary activation immediately after NMES and RT [28] in healthy subjects. Conroy *et al.* have shown that patients with knee OA have significantly reduced torque per unit of quadriceps area compared to controls, despite no difference in quadriceps strength or quadriceps size [29]. This reflects poorer muscle quality in knee OA. Increases in quadriceps size in this cohort may therefore produce less than expected increases in torque. Further work using electromyography should clarify our surprising finding.

An important limitation of NMES is the limited spatial recruitment of muscle fibres [20]. The novel garment stimulator used in this study utilises 2 channels and a “multipath” system of stimulation and is purported to achieve greater muscle recruitment. In the current study, we did not analyse the CSA of the component muscles of the QFM separately. It would be worthwhile in future work to do so in order to identify differences in muscle hypertrophy induced by the two training modalities.

Adherence is an important predictor of clinical outcome in response to exercise training in patients with knee OA [5]. Factors that have been identified to improve adherence include educating patients of the benefits resulting from participation in an exercise program, simplifying exercise regimens, setting clear and attainable goals, providing social interaction and providing regular follow-up. Although we found an 8% higher adherence rate in the NMES group than the RT group, the difference was not significant. Possible factors accounting for the high adherence rate in the NMES group include the relative simplicity of the NMES training routine, the novelty of the modality and participant knowledge that the device was logging usage data.

Chamberlain *et al.* compared a home exercise based program to hospital-based physical therapy in patients with knee OA and found similar improvements in function, pain and strength in response to 4 weeks of therapy [30]. Patients who were notified after the intervention of a further assessment at 12 weeks, continued their daily exercises, and maintained their improvements in pain, function and strength. In contrast, patients who were not notified of further testing were more likely to cease exercising and experience more pain.

We concur that regular follow-up is essential to achieve benefits and good adherence with home-based therapy. The supervision of two-thirds of the home-based RT sessions no doubt contributed to good adherence in the present study. A feature of the NMES device is the capacity to keep an electronic log of usage. The log provides a means to monitor adherence to unsupervised home therapy, and probably played a role in incentivising patients to adhere to the training program.

Despite the lack of supervision, the NMES group showed a non-significantly higher adherence rate than the RT group. Given the resource-intensiveness and cost of traditional physical therapy, home-based NMES offers an attractive alternative.

Limitations of this study include the limited sample size, the short follow-up period, the lack of a placebo intervention for the control group and the absence of measurements of voluntary activation such as electromyography or twitch interpolation.

## Conclusions

A six-week home-based, unsupervised NMES program significantly improves functional performance in patients with moderate to severe knee OA, and is an acceptable alternative to resistance training in this patient cohort. We found significant intra-group increases in quadriceps CSA for both training groups, but improvements in quadriceps strength and in self-reported disability scores did not reach significance relative to the control group.

Dose–response studies with longer follow-up are required to establish the optimum frequency and intensity of NMES training.

## Competing interests

The authors declare that they have no competing interests.

## Authors’ contributions

RBB was the principle researcher involved in study conception and design, as well as implementation, analysis and interpretation of data, and manuscript preparation. RW made substantial contributions to the conception and design of the study and the drafting of the article. JCO made substantial contributions to the conception and design of the study and the revision of the article. BE made substantial contributions to the acquisition of data and the drafting of the article. JOB made substantial contributions to the conception and design of the study and the revision of the article. NM made substantial contributions to the conception and design of the study, the analysis and interpretation of the data and the revision of the article. All authors read and approved the final manuscript.

## Pre-publication history

The pre-publication history for this paper can be accessed here:

http://www.biomedcentral.com/1471-2474/13/118/prepub
